# Polysialic Acid Acute Depletion Induces Structural Plasticity in Interneurons and Impairs the Excitation/Inhibition Balance in Medial Prefrontal Cortex Organotypic Cultures

**DOI:** 10.3389/fncel.2016.00170

**Published:** 2016-06-29

**Authors:** Esther Castillo-Gómez, Marta Pérez-Rando, Sandra Vidueira, Juan Nacher

**Affiliations:** ^1^Neurobiology Unit/BIOTECMED, Cell Biology Department, Universitat de ValènciaValencia, Spain; ^2^Centro de Investigación Biomédica en Red de Salud Mental (CIBERSAM): Spanish National Network for Research in Mental HealthMadrid, Spain; ^3^Fundación Investigación Hospital Clínico de Valencia, INCLIVAValencia, Spain

**Keywords:** E/I balance, PSA-NCAM, neuronal structural plasticity, mPFC cultures, schizophrenia, major depression

## Abstract

The structure and function of the medial prefrontal cortex (mPFC) is affected in several neuropsychiatric disorders, including schizophrenia and major depression. Recent studies suggest that imbalances between excitatory and inhibitory activity (E/I) may be responsible for this cortical dysfunction and therefore, may underlie the core symptoms of these diseases. This E/I imbalance seems to be correlated with alterations in the plasticity of interneurons but there is still scarce information on the mechanisms that may link these phenomena. The polysialylated form of the neural cell adhesion molecule (PSA-NCAM) is a good candidate, because it modulates the neuronal plasticity of interneurons and its expression is altered in schizophrenia and major depression. To address this question, we have developed an *in vitro* model using mPFC organotypic cultures of transgenic mice displaying fluorescent spiny interneurons. After enzymatic depletion of PSA, the spine density of interneurons, the number of synaptic puncta surrounding pyramidal neuron somata and the E/I ratio were strongly affected. These results point to the polysialylation of NCAM as an important factor in the maintenance of E/I balance and the structural plasticity of interneurons. This may be particularly relevant for better understanding the etiology of schizophrenia and major depression.

## Introduction

The medial prefrontal cortex (mPFC) is one of the most complex and highly developed neocortical regions, whose structure and function are profoundly affected in different neuropsychiatric disorders, including schizophrenia and major depression (Lewis, 2012; Price and Drevets, 2012). While some preclinical and clinical studies suggest that the dysfunction of the mPFC found in these disorders is mainly associated with alterations in glutamatergic neurotransmission and in the structure of pyramidal neurons (Sanacora et al., 2012; Glausier and Lewis, 2013), many others suggest that deficits in GABAergic neurotransmission, particularly on the perisomatic innervation of pyramidal neurons, are mainly responsible for this malfunction (Luscher et al., 2011; Inan et al., 2013). However, whether alterations in the structure of mPFC interneurons are also associated with the pathophysiology of schizophrenia and major depression, remains under research.

During the last years, our laboratory and others have found that the polysialylated form of the neural cell adhesion molecule (PSA-NCAM) plays a key role in the structural plasticity of neurons, allowing the presence of plastic events such as dendritic, spine and synaptic remodeling (Bonfanti, 2006; Nacher et al., 2013; Podestá et al., 2014). Moreover, alterations in the expression of this molecule have been found in the mPFC of patients suffering from major depression and schizophrenia and in animal models of these disorders (Barbeau et al., 1995; Vicente et al., 1997; Isomura et al., 2011; Gilabert-Juan et al., 2013; Nacher et al., 2013; Wędzony et al., 2013). Since in this region, both in rodents and humans, PSA-NCAM is exclusively expressed by mature interneurons (for review see Bonfanti, 2006; Nacher et al., 2013), we can hypothesize that alterations in the expression of this molecule may affect the structure and connectivity of interneurons in these disorders.

Moreover, recent studies suggest that alterations on the precise balance between excitatory and inhibitory activity (E/I) in the mPFC (E/I balance), without the existence of correct compensatory mechanisms, may constitute the basis of the cognitive and social deficits frequently found in schizophrenia and major depression (Hasler et al., 2007; Marín, 2012; Inan et al., 2013). Furthermore, alterations in E/I ratio seem to be correlated with impairments in brain plasticity (Hensch and Bilimoria, 2012; Inan et al., 2013). Since PSA-NCAM is expressed in inhibitory elements (Nacher et al., 2013), it may have a potential role in the maintenance of this E/I balance.

In order to develop an *in vitro* system in which we could analyze the role of PSA-NCAM in the structure and connectivity of mPFC inhibitory neurons and in the maintenance of E/I balance, we enzymatically removed PSA from NCAM in organotypic cultures from mice-expressing the enhanced green fluorescent protein (EGFP) under the glutamic acid decarboxylase (GAD) promoter, which labels the complete morphology of a subpopulation of spiny interneurons (Oliva et al., 2000; Guirado et al., 2014a; Nacher et al., 2013). Forty-eight hours after PSA depletion, we analyzed the effects on the dendritic spine density of GAD-EGFP expressing interneurons, the E/I ratio in the neuropil and the number of synaptic puncta surrounding pyramidal neuron somata.

## Materials and Methods

### Animals

Transgenic mice pups [GIN, FVB- Tg(GadEGFP)45704Swn] (P8) were used for all experimental procedures. These animals express the EGFP under the promoter of the GAD gene, which codifies the enzyme responsible for the synthesis of the inhibitory neurotransmitter GABA. Adult mice were purchased from Jackson laboratories (Bar Harbor, Maine, ME, USA) and mice pups were obtained in our animal facility.

Pregnant mice were housed alone in a temperature- and humidity-controlled environment and maintained on a 12 h light/dark cycle with food and water available *ad libitum*. Mice pups were kept with their mothers until being used.

All animal experimentation was conducted in accordance with the Directive 2010/63/EU of the European Parliament and of the Council of 22 September, 2010 on the protection of animals used for scientific purposes and was approved by the Committee on Bioethics of the Universitat de València. Every effort was made to minimize the number of animals used and their suffering.

### Medial Prefrontal Cortex Slice Cultures

#### Preparation of Slice Cultures

P8 transgenic mice pups were decapitated and their brains were removed from the skull under sterile conditions. Brains were placed into Petri dishes filled with cold (4°C) sterile dissecting medium [1% glucose (0, 5 gr/mL, MERCK), 0, 2% penicillin/streptomycin, 0, 1% hexamycin, 0, 5% L-glutamin, 0, 05% fungizone (GIBCO) in GBSS (Sigma-Aldrich)]. The overlying pia was gently removed and coronal cuts were made to remove portions of the rostral and caudal poles, leaving the frontoparietal region intact. The right and left cortices were cut simultaneously in the coronal plane at a thickness of 350 μm with a McIlwain^TM^ Tissue Chopper. The slices obtained were transferred into dissecting medium and separated gently by agitation. Slices containing mPFC were placed on moistened translucent membranes of tissue culture inserts (0.4 μm, Millicell-CM, Millipore, Bedford, MA, USA) and immersed in 1 mL of Serum-OPTIMEM culture medium [25% heat inactivated horse serum, 25% HBSS, 50% Optimem-1 (GIBCO) supplemented with 10 μL/mL glucose (0, 5 gr/mL, MERCK)]. Three slices were cultured in the same insert and six inserts were placed together in six-well plates. To ensure that slices from the control and the treated groups were cultured under identical conditions, three inserts from each plate were designated as control group and the other three, as treated group. Cultures were stored in a humid atmosphere at 36°C, in 5% CO_2_ for 14 days (HERAcell^®^ 150i, Thermo Scientific) and medium was changed twice a week.

#### Delivery of Endo-N or Vehicle Solution

After 12 days *in vitro* (DIV 12), 2 U/mL of the enzyme Endo-N (AbCys-Eurobio, Paris, France) was added to the culture medium, as described previously by Di Cristo et al. (2007). Organotypic cultures from control group received the same amount of vehicle solution (glycerol) in their medium. Two days after (DIV 14) slices were fixed as described below.

#### Histological Processing of Slices

Cultures were fixed by immersing the inserts for 30 min in a solution of 4% paraformaldehyde in 0.1 M phosphate buffer (PB; pH 7.4) and then washed with PB intensively. Because pilot experiments had demonstrated that our mPFC slices decreased their thickness to 150–200 μm, tissue was not cut into thinner sections after its fixation.

In order to separate the slices that had been cultured in the same insert, insert membranes were cut surrounding each slice and then slices were stored at 4°C fully immersed in PB.

### Immunohistochemistry

Tissue slices were processed free-floating as follows: briefly, slices were incubated for 1 min in an antigen unmasking solution (0.01 M citrate buffer, pH 6) at 100°C. After cooling down sections to room temperature, the endogenous peroxidase was blocked with 10 min incubation in a solution of 3% H_2_O_2_ in phosphate buffered saline (PBS). Afterwards, slices were incubated in 10% normal donkey serum (NDS; Jackson Immunoresearch) in PBS with 0.2% Triton-X100 (Sigma-Aldrich, St. Louis, MO, USA) for 1 h. Then, they were incubated overnight at room temperature with mouse monoclonal IgG anti-neuronal nuclear antigen (NeuN, 1:100; Chemicon-Millipore) or mouse monoclonal anti-PSA-NCAM IgM antibody (1:700; AbCys-Eurobio). After washing, sections were incubated for 1 h with biotinilated donkey anti-mouse IgG or donkey anti-mouse IgM secondary antibodies (1:400; Jackson Immunoresearch), followed by an avidin-biotin-peroxidase complex (ABC; Vector Laboratories) for 1 h in PBS. Color development was achieved by incubating with 3, 3′-diaminobenzidine tetrahydrochloride (DAB; Sigma-Aldrich) and 0.033% H_2_O_2_ for 4 min. PBS containing 0.2% Triton-X100 and 3% NDS was used for primary and secondary antibodies dilution. All sections passed through all procedures simultaneously to minimize any difference from immunohistochemical staining itself. Sections were finally mounted on slides, dried for 1 day at room temperature, dehydrated with ascending alcohols and rinsed in xylene. After this, sections were coverslipped using Eukitt mounting medium (Sigma-Aldrich).

For double and triple fluorescence immunohistochemistry, tissue was processed as described above but omitting the endogenous peroxidase blockade. Slices were incubated overnight at room temperature with the following cocktails of primary antibodies: (1) Chicken anti-GFP (Chemicon-Millipore, 1:1000) and mouse IgM anti-PSA-NCAM; (2) Chicken anti-GFP, mouse IgG2b anti-calcium/calmodulin-dependent kinase II-α (CaMKII-α; Abcam, 1:500) and rabbit anti-vesicular GABA transporter (VGAT; Synaptic Systems, 1:1000); (3) Chicken anti-GFP, mouse IgG anti-CaMKII-α and rabbit anti-synaptophysin (SYN; Chemicon-Millipore, 1:1000); (4) Chicken anti-GFP, mouse IgG anti-CaMKII-α and rabbit anti-parvalbumin (PV; Swant, 1:2000); (5) Chicken anti-GFP, mouse IgG anti-CaMKII-α and guinea pig anti-vesicular glutamate transporter 1 (VGLUT1; Chemicon-Millipore, 1:2000); (6) Mouse IgM anti-PSA-NCAM, mouse IgG2b anti-CaMKII-α and rabbit anti-calretinin (CR; Swant, 1:1000); and (7) Mouse IgM anti-PSA-NCAM, guinea pig anti-PV (Synaptic Systems, 1:4000) and rabbit anti-calbindin (CB; Swant, 1:1000). After washing, sections were incubated for 1 h with five different cocktails of fluorescent secondary antibodies: (1) Donkey anti-chicken Dylight 488 (Jackson Immunoresearch, 1:400) and donkey anti-mouse IgM Dylight 555 (Jackson Immunoresearch, 1:400); (2) Donkey anti-chicken Dylight 488, mouse IgG Dylight 647 (Jackson Immunoresearch, 1:400) and donkey anti-rabbit Dylight 555 (Jackson Immunoresearch, 1:400); (3) Donkey anti-chicken Dylight 488, donkey anti-mouse IgG Dylight 647 and goat anti-guinea pig Dylight 549 (Jackson Immunoresearch, 1:400); (4) Donkey anti-mouse IgG2b Dylight 405 (Jackson Immunoresearch, 1:400), donkey anti-mouse IgM Dylight 549 (Jackson Immunoresearch, 1:400) and donkey anti-rabbit Dylight 649 (Jackson Immunoresearch, 1:400); and (5) Donkey anti-mouse IgM Dylight 549, goat anti-guinea pig Dylight 649 (Jackson Immunoresearch, 1:400) and donkey anti-rabbit Alexa Fluor 405 (Jackson Immunoresearch, 1:400). Finally, sections were washed, mounted on slides and coverslipped using DakoCytomation fluorescent mounting medium (Dako North America Inc., Carpinteria, CA, USA).

### Analysis of Neuropil Puncta Density and Calculation of the E/I Ratio

The density of neuropil puncta expressing GAD-EGFP, VGAT, VGLUT1 or SYN in the mPFC was analyzed following the same protocol described before Guirado et al. (2014b). That is, from each immunostaining, slices from the same rostral-caudal level were examined under a confocal microscope (Leica TCS SPE) using a 63× oil objective and 2× digital zoom magnification. Confocal *z*-stacks covering the whole depth of the slices were taken with 1 μm step size and only subsets of confocal planes with the optimal penetration level for each antibody were selected. On these planes, five small regions of neuropil (300 μm^2^) from deep layers of the mPFC (layers V and VI) were selected for analysis, in order to avoid blood vessels and cell somata. Images were processed using FIJI-ImageJ Software as follows: the background fluorescence was subtracted with a rolling value of 50, converted to 8-bit deep images and binarized using a determined threshold value. This value depended on the marker and was kept the same for all images with the same marker. Then, the images were processed with a blur filter to reduce noise and separate closely apposed puncta. Finally, the number of the resulting dots per region was automatically counted and divided into three size groups: from 4 to 22 pixels, from 23 to 33 pixels and from 34 to infinite. Only puncta from the first and second group were taken into account, since puncta from the third group only represented fibrillar processes. Puncta density means from these five squared areas were determined and the resulting values were subjected to unpaired Student’s *t*-test with the number of slices as the *n*. For the calculation of the E/I ratio in the neuropil, the density of excitatory puncta (VGLUT1+) was divided by the density of inhibitory puncta (VGAT+) and the resulting values were subjected to unpaired Student’s *t*-test with the number of slices as the *n*.

### Quantification of Perisomatic Puncta on Medial Prefrontal Cortex Pyramidal Neurons and Calculation of the E/I Ratio in the Perisomatic Region

The density of puncta expressing GAD-EGFP, VGAT, VGLUT1, SYN or PV surrounding pyramidal neuron somata (identified by CaMKII-α expression) was analyzed following a previously described protocol (Guirado et al., 2014b). In brief, six CaMKII-α expressing neurons from the deep layers of mPFC, which include layer V but not layer III, and displaying triangular-shaped soma were randomly selected from each slice. Confocal *z*-stacks covering the whole depth of the neuron somata were taken with 0.5 μm step size using a 63× oil objective with 2× digital zoom magnification (Leica TCS SPE confocal microscope). Stacks were processed with LSM 5 Image Browser software at 4× -Zoom magnification. The profile of the soma of these neurons was drawn and puncta localized within an area 0.5 μm distal from the edge of this profile were analyzed. A puncta was defined as a structure displaying an area not smaller than 0.15 μm^2^ and not larger than 2.5 μm^2^ (Di Cristo et al., 2007). The density of puncta (number of puncta per micron of soma perimeter) was analyzed on five consecutive confocal planes from each selected neuron, in which the penetration of each antibody was optimal. Means were determined and the resulting values were subjected to unpaired Student’s *t*-test with the number of slices as the *n*. For the calculation of E/I ratio in the perisomatic region, the density of excitatory puncta (VGLUT1+) was divided by the density of inhibitory puncta (VGAT+) and the resulting values were subjected to unpaired Student’s *t*-test with the number of slices as the *n*.

### Analysis of Dendritic Spine Density in Cultured GAD-EGFP Expressing Interneurons

Dendritic spine density of GAD-EGFP expressing interneurons was analyzed following a previously described protocol (Guirado et al., 2014a). These interneurons have been previously identified as somatostatin expressing Martinotti cells in the adult mPFC (Oliva et al., 2000). From each slice, six EGFP-expressing neurons located in mPFC layer V were examined by confocal microscopy at 63× magnification. In order to be analyzed, neurons and dendrites had to fulfill the following features: (1) the measured dendrite must not be truncated; (2) the dendrite measured must be longer than 180 μm, and (3) the soma must be located at least 30 μm deep from the surface of the tissue. These cells were first identified using conventional fluorescence microscopy and then, confocal *z*-stacks covering all its three-dimensional extension were taken with 0.1 μm step size. Stacks were processed with LSM 5 Image Browser software at 4× -Zoom magnification and spines were quantified in three successive segments of 60 μm distances up to a total length of 180 μm. The subdivision of the dendritic length has been chosen because previous reports from our laboratory have found differences in the dendritic spine density along their dendrites, at least in the hippocampus (Guirado et al., 2014a). Moreover, Endo-N depletion, as well as fluoxetine treatment, induces differential effects on the dendritic spine density in these different fragments (Guirado et al., 2014a,b). Spine density values (overall or per segment) were expressed as number of spines/μm length. Means were determined and the resulting values were analyzed by unpaired Student’s *t*-test with the number of slices as the *n*.

## Results

### Microscopic Architecture and PSA-NCAM Expression in Organotypic Cultures from the Medial Prefrontal Cortex of GAD-EGFP Expressing Mice

**Figure 1:**
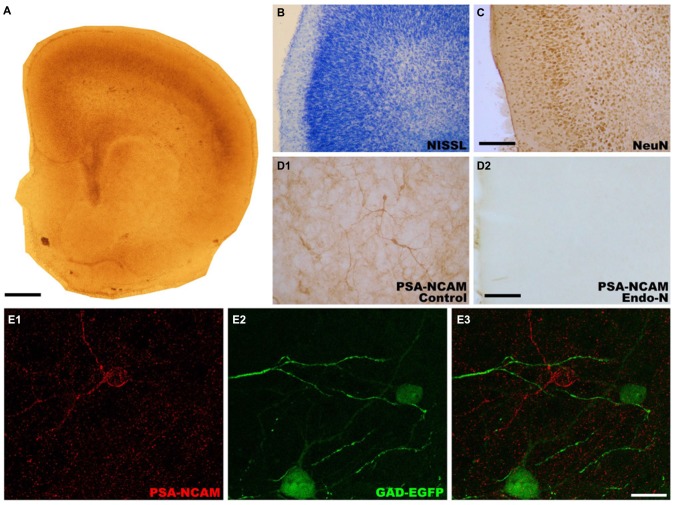
**Photographs showing the microscopic architecture and the pattern of polysialylated form of the neural cell adhesion molecule (PSA-NCAM) expression in organotypic cultures of the medial prefrontal cortex (mPFC). (A)** Panoramic view of a mPFC slice after 14 days *in vitro*. **(B,C)** Microphotographs showing that the layered organization of neurons in the mPFC is preserved after 14 days *in vitro*. **(B)** Nissl stained slice. **(C)** Slice immunostained for NeuN, a marker of mature neurons, **(D)** PSA-NCAM expression is observed in neuronal somata, neurites and neuropil in control slices **(D1)**, but it is completely absent in Endo-N treated-slices **(D2)**. **(E)** Glutamic acid decarboxylase (GAD)-enhanced green fluorescent protein (EGFP) expressing interneurons in control slices do not co-express PSA-NCAM. Scale bar: 10 μm for **(A)** 25 μm for **(B)** and **(C)** 100 μm for **(D1,D2)**, 20 μm for **(E1–E3)**.

**Figure 2:**
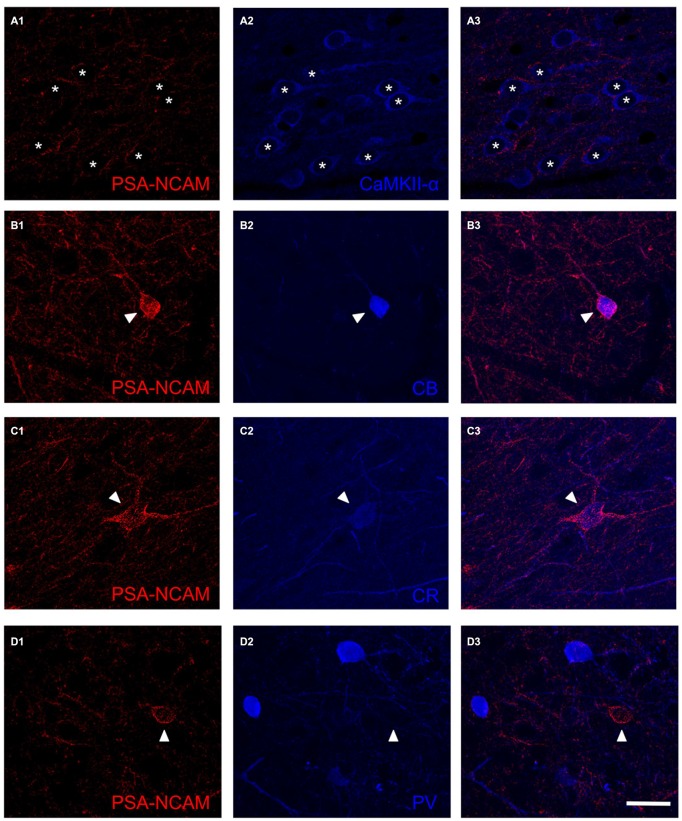
**Confocal microscopy images showing the expression of PSA-NCAM (A1,B1,C1,D1), a marker for pyramidal neurons (CamKII-α; A2) and several markers for interneurons: calbindin (CB; B2) calretinin (CR; C2) and parvalbumin (PV; D2).** Note the expression of PSA-NCAM in the soma of interneurons expressing CB and CR (**B3**,**C3**, arrowheads) but not in the soma of pyramidal neurons (**A3**, arrowhead) or interneurons expressing PV (**D3**, arrowhead). In the neuropil, PSA-NCAM expression can be found in perisomatic boutons surrounding pyramidal neurons (**A3**, asterisks) and also in dendrites and puncta expressing markers for interneurons **(B3,C3,D3)**. Scale bar: 20 μm.

In order to study whether the mPFC conserved its general appearance after 14 DIV (Figure 1A), we performed Nissl staining with toluidine blue (Figure 1B) and NeuN immunohistochemistry (Figure 1C). These staining revealed that the gross microscopic architecture of the mPFC was maintained in the cultures as observed *in vivo*. PSA-NCAM expression was observed in neuronal somata and neuropil in control slices (Figures 1D1,E1), but it was completely absent in Endo-N treated slices (Figure 1D2). As has been described in adult mice ((Nacher et al., 2013) for review*)*, the somata of GAD-EGFP expressing neurons in mPFC organotypic cultures never co-expressed PSA-NCAM (Figures 1E1–E3). However, while PSA-NCAM expression is absent from the somata of pyramidal cells (labeled with CAMKII-α antibody, Figure 2A3), it is present in the somata of interneurons expressing calbindin (Figure 2B3) or calretinin (Figure 2C3).

### Effects of Polysialic Acid Depletion on the Expression of Synaptic Markers in the Neuropil

#### Endo-N Decreases the Density of Neuropil Puncta Expressing Inhibitory Markers and Increases the Density of Neuropil Puncta Expressing Excitatory and Synaptic Markers

**Figure 3:**
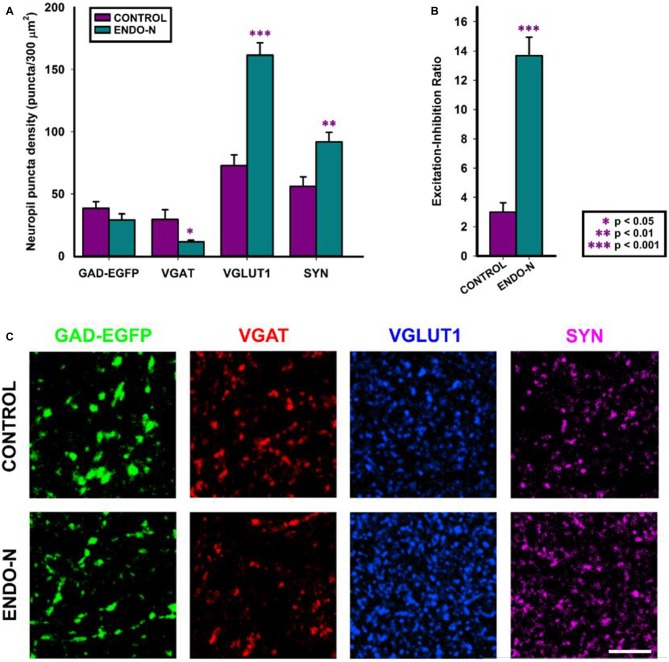
**Confocal analysis of GAD-GFP, vesicular GABA transporter (VGAT), vesicular glutamate transporter 1 (VGLUT1) and synaptophysin (SYN) expression in the neuropil of mPFC organotypic cultures. (A)** Graph representing neuropil puncta density (number of puncta/300 μm^2^) in control (purple bar) and Endo-N treated cultures (turquoise bar) for the different markers shown in **(C)**. Note that statistically significant changes (unpaired Student’s *t*-test) were found in the density of VGAT, VGLUT1 and SYN expressing puncta. **(B)** Graph showing statistically significant changes (unpaired Student’s *t*-test) in the Excitation/Inhibition ratio (VGLUT1/VGAT neuropil puncta density) after Endo-N treatment. **(C)** Confocal images (single confocal planes) comparing the expression of the different proteins analyzed between control and Endo-N treated cultures. Scale bar 5 μm. *p-value < 0.05; **p-value < 0.01; ***p-value < 0.001.

In order to know whether the depletion of PSA from NCAM had an impact on the expression of different markers of excitatory, inhibitory and general synaptic markers, we have performed immunohistochemical staining with antibodies directed specifically to different synaptic proteins. The regions for the analysis of neuropil were selected from microphotographs of the deep layers (V and VI) of the mPFC (see “Materials and Methods” Section). PSA depletion induced a statistically significant decrease in the density of puncta expressing vesicular GABA transporter (VGAT) in the mPFC neuropil (*t*_(15)_ = 2.398; *p* = 0.030). No statistically significant differences were found when analyzing GAD-EGFP expressing puncta (*t*_(15)_ = 1.297; *p* = 0.214), although a trend towards a decrease was observed (Figures 3A,C). Statistically significant increases in the density of vesicular glutamate transporter 1 (VGLUT1) expressing puncta were observed after 2 days of Endo-N delivery in the mPFC neuropil (*t*_(15)_ = −6.621; *p* < 0.001; Figures 3A,C). Endo-N treatment induced a statistically significant increase in the density of synaptophysin (SYN) expressing puncta in the mPFC neuropil (*t*_(15)_ = −3.300; *p* = 0.005; Figures 3A,C).

#### Endo-N Impairs E/I Ratio in the Medial Prefrontal Cortex Neuropil

To investigate whether Endo-N treatment affected the proportion of puncta expressing excitatory synaptic markers vs. those expressing inhibitory ones, we analyzed the ratio between excitatory and inhibitory vesicular transporter expressing puncta (VGLUT+/VGAT+). To this end, we have analyzed the density of puncta on selected small regions that lacked neuronal somata and blood vessels. We found that this ratio was markedly increased 2 days after Endo-N delivery (*t*_(13)_ = −7.202; *p* < 0.001; Figures 3B,C).

### Effects of Endo-N Treatment on the Perisomatic Puncta on Medial Prefrontal Cortex Pyramidal Neurons

#### Endo-N Increases the Density of Perisomatic Puncta Expressing Inhibitory, Excitatory and Synaptic Markers

**Figure 4:**
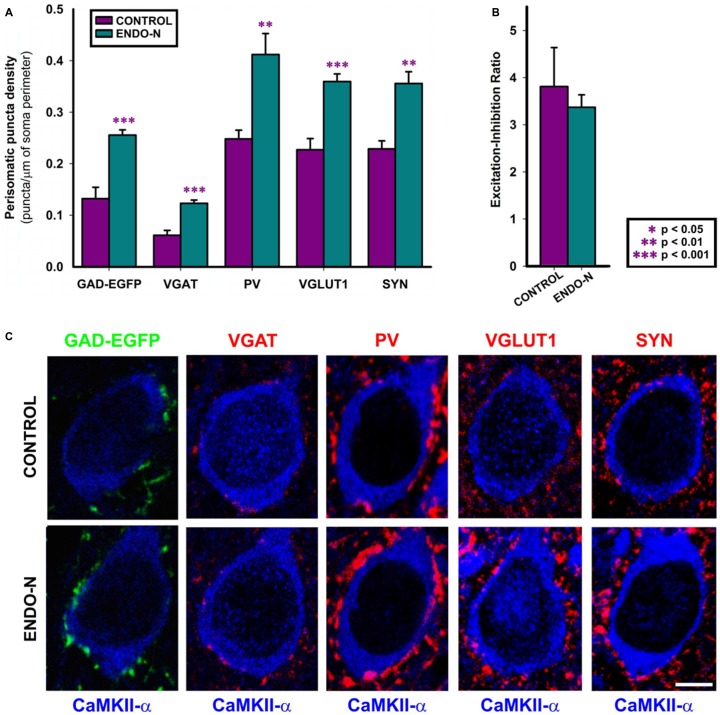
**Confocal analysis of GAD-GFP, VGAT, PV, VGLUT1 and SYN expression in the perisomatic region of pyramidal neurons. (A)** Graph representing statistically significant changes (unpaired Student’s *t*-test) in perisomatic puncta density (number of puncta/μm of soma perimeter) after Endo-N treatment for the all the markers shown in **(C)**. **(B)** Graph representing the Excitation/Inhibition ratio (VGLUT1/VGAT perisomatic puncta density) between control (purple bar) and Endo-N treated cultures (turquoise bar). No statistically significant differences were found after unpaired Student’s *t*-test. **(C)** Confocal images (single confocal planes) comparing the density of the puncta expressing the different proteins between control and Endo-N treated cultures. Scale bar 5 μm. **p-value < 0.01; ***p-value < 0.001.

Since previous results from our laboratory have shown that the long-term depletion of PSA *in vivo* (2 weeks) increased the density of puncta expressing inhibitory synaptic markers around the somata of mPFC pyramidal cells (Castillo-Gómez et al., 2011), we have studied this parameter on organotypic cultures in order to see whether Endo-N produces similar results. The analysis of perisomatic puncta has been performed in deep layers of the mPFC, which included layer V. The regions selected for the analysis of perisomatic puncta were identified by the presence of CAMKII positive pyramidal neurons. Short-term PSA depletion *in vitro* (2 days) increased the density of inhibitory puncta surrounding the somata of pyramidal neurons. Statistically significant increases were found in the density of GAD-EGFP (*t*_(15)_ = −5.309, *p* < 0.001), VGAT (*t*_(15)_ = −5.547, *p* < 0.001) and PV (PV, *t*_(14)_ = −3.699, *p* = 0.002) expressing puncta (Figures 4A,C). The density of VGLUT1 expressing puncta in the perisomatic region of pyramidal neurons increased 2 days after the delivery of Endo-N (*t*_(15)_ = −5.547, *p* < 0.001; Figures 4A,C). Short term PSA depletion also induced a statistically significant increase in the density of SYN expressing puncta surrounding the somata of pyramidal neurons (*t*_(12)_ = −4.582, *p* < 0.0001), similar to what we observed *in vivo* after long-term PSA depletion (Castillo-Gómez et al., 2011; Figures 4A,C).

#### Endo-N Does Not Change E/I Ratio in the Perisomatic Region of Pyramidal Neurons

In contrast to what we observed when taking into account the whole neuropil of the mPFC, the ratio between excitatory and inhibitory vesicular transporter-expressing puncta (VGLUT+/VGAT+) in the perisomatic region of pyramidal neurons was maintained in Endo-N treated slices (*t*_(15)_ = 0.680; *p* = 0.507; Figures 4B,C).

### Changes in the Dendritic Spine Density of mPFC Interneurons after PSA Depletion

Before analyzing the dendritic spine density of GAD-GFP expressing interneurons from mPFC organotypic cultures, we performed a morphological analysis of these neurons. This analysis revealed that all of them were multipolar cells and that none of them presented a bipolar morphology. Consequently, and also taking into account that these cells are somatostatin+, they can be classified as Martinotti interneurons, in accordance with our previous report on the adult mPFC of this mice strain (for review see Nacher et al., 2013).

**Figure 5:**
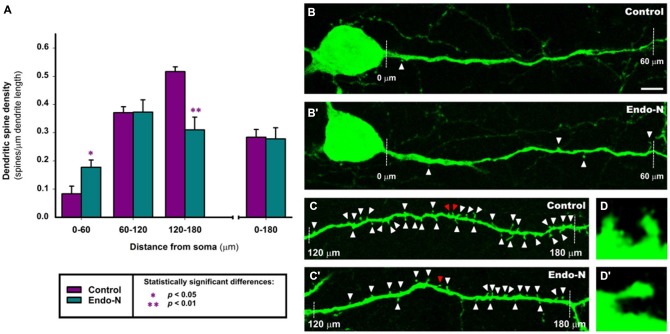
**Confocal analysis of dendritic spine density in GAD-EGFP expressing interneurons of mPFC organotypic cultures. (A)** Graph comparing the dendritic spine density (spines/μm of dendrite length) of GAD-EGFP expressing interneurons from mPFC slices treated with Endo-N with those from slices treated with vehicle solution (control). 0–60, 60–120 and 120–180 indicate dendrite segments of 60 μm located in 0–60, 60–120 and 120–180 μm segments from the interneuron soma, respectively. Asterisk in bars indicate statistically significant differences in unpaired Student *t*-test: *p < 0.05, **p < 0.01. **(B–D′)** Confocal microscopic images of GAD-EGFP expressing interneurons in mPFC organotypic cultures treated with vehicle solution (control; **B–D**) or Endo-N **(B′–D′)**. Note the slight increase in the density of spines after Endo-N treatment in the 0–60 μm segment **(A,B)** but the marked decrease in this density in the 120–180 μm segment **(A,C)**. Images **(D,D′)** are 10X enlargements of the spines marked with red arrowheads in **(C,C′)**, respectively. Scale bar: 5 μm for **(B,B′,C,C′)**; 50 μm for **(D,D′)**.

The analysis of dendritic spine density showed that PSA depletion for 2 days had an intense effect on the density of these postsynaptic structures. The dendrites of GAD-EGFP expressing interneurons were divided into three successive segments of 60 μm length up to a total length of 180 μm (see “Materials and Methods” Section). We found statistically significant differences in dendritic spine density in the most proximal segment to the soma (*t*_(10)_ = −2.460, *p* = 0.034; 112.6% increase; Figures 5A–B′) and in the most distal segment (*t*_(8)_ = 4.289, *p* = 0.003; 39.8% decrease; Figures 5A,C,C′,D,D′). No statistically significant differences were found when analyzing the medial segment (*t*_(10)_ = −0.471, *p* = 0.648) or when taking into account the whole length of the dendritic fragment studied (*t*_(11)_ = 0.121, *p* = 0.906; Figure 5A).

## Discussion

In the present study we have shown that the organotypic cultures of the mPFC are a valid model to reproduce *in vitro* the inhibitory networks of this cortical region and their structural plasticity. These cultures display PSA-NCAM immunoreactive interneurons similar to those found in adult animals (for review see Bonfanti, 2006; Nacher et al., 2013). The specific depletion of PSA from NCAM induces changes similar to those observed after this treatment *in vivo*, decreasing the expression of markers of inhibitory neurotransmission in the neuropil and increasing the density of inhibitory perisomatic puncta around pyramidal neurons (Castillo-Gómez et al., 2011). Interestingly, the number of puncta expressing molecules related to excitatory neurotransmission is also increased in the neuropil and the perisomatic region of pyramidal neurons. Moreover, this report describes for the first time that this treatment also affects the E/I ratio and profoundly alters the structure of mPFC inhibitory neurons.

### Validity of Medial Prefrontal Cortex Organotypic Cultures and of PSA Depletion

The mPFC organotypic cultures used in this study constitute a valid approach to reproduce the intrinsic connectivity of this cortical region, especially that involving local inhibitory circuits. We have observed that the cytoarchitectonic organization of the cultures closely resembles that of the adult mPFC. Previous studies have shown that the physiological properties of both pyramidal neurons and interneurons in organotypic cultures are very similar to those of neurons characterized *in vivo* or in acute slice preparations (Plenz and Aertsen, 1996a; Klostermann and Wahle, 1999; Hinterhoelzl et al., 2003). These cultures display a well-balanced state of excitation and inhibition, suggesting that intrinsic cortical mechanisms are sufficient for the expression of cell-type-specific electrophysiological properties and persistent activity (Plenz and Aertsen, 1996b).

The time course of PSA depletion appears to be similar in the adult mPFC and in organotypic cultures. Although we have not studied the effects of this depletion in the mPFC for periods shorter than 2 weeks *in vivo*, we know that when the enzyme is injected intracerebrally in the primary somatosensory cortex above the hippocampus most PSA is depleted from this neocortical region and the dorsal hippocampus after 2 days (Guirado et al., 2014a). Moreover, in hippocampal organotypic cultures we have observed a complete depletion of PSA 24 h after Endo-N treatment (Guirado et al., 2014a). Consequently, this depletion in organotypic cultures provides a very good model for studying the role of PSA-NCAM on mPFC inhibitory networks.

### Effects of PSA-NCAM Depletion on Excitatory and Inhibitory Circuits of the Medial Prefrontal Cortex

Although previous results from our laboratory using long term PSA depletion (14 days) from the mPFC *in vivo* have shown that this treatment does not induce changes in GAD67 or SYN expression in the neuropil (Castillo-Gómez et al., 2011), we have found that 2 days of Endo-N treatment in mPFC organotypic cultures induced significant changes in the expression of VGAT (decrease), SYN and VGLUT1 (increases). Surprisingly, despite the reduction observed in the density of VGAT expressing puncta in the neuropil, we have not detected changes in those expressing GAD-EGFP. Although there is a trend towards a decrease in the density of these puncta, the lack of significant differences may be due to the fact that these structures only belong to a subtype of interneurons in this strain of transgenic mice, the Martinotti cells (Oliva et al., 2000; Guirado et al., 2014a,b). It is possible that the axonal projection of these interneurons was not affected by the Endo-N treatment.

We believe that this differential effect *in vivo* vs. *in vitro* of Endo-N in the expression of these markers may be due to the different degree of maturation of both excitatory and inhibitory synapses. Probably these synapses are less mature in the organotypic cultures and they still express high levels of PSA-NCAM. Although the expression of PSA-NCAM is very low in the somata of pyramidal neurons of the mPFC cultures, the high density of PSA-NCAM expression in the neuropil indicates that this molecule may be expressed in a considerable proportion of synapses, which, after PSA depletion, may mature as both excitatory and inhibitory synapses. In fact, although PSA-NCAM is exclusively located in inhibitory elements in the adult mPFC, it is expressed by immature excitatory neurons, such as recently generated granule neurons in the adult hippocampus (Seki and Arai, 1993). The depletion of PSA from NCAM may also promote the differentiation of immature excitatory synapses in the cultures, leading to the increase in VGLUT1 and SYN expression detected in our experiment. A previous report has described that the perinatal depletion of PSA with Endo-N highly increases the number of mossy fiber synapses (the terminal synaptic boutons of granule neurons) in mice (Seki and Rutishauser, 1998). This putative increase in the number of excitatory synapses may be the responsible for the alteration in the E/I ratio that we observe in the neuropil of the mPFC organotypic cultures.

### Changes in the Dendritic Spine Density of Martinotti Interneurons

We have found that Endo-N treatment induced a significant increase in dendritic spine density in the most proximal segment to the soma and a significant reduction in the most distal segment. Although the increase in spine density in the proximal segment of the dendrites is much higher than that observed in the distal portion, it has to be noted that the density of spines in the proximal segment of these interneurons is considerable lower than in the distal segment, both in adult animals (Guirado et al., 2014a,b) and in organotypic cultures (present results). A previous study found that Endo-N induced a significant increase in spine density on the distal segment of hippocampal EGFP expressing interneurons in animals treated for 2 days *in vivo*, whereas this density was decreased when the treatment was prolonged for 7 days (Guirado et al., 2014a). In this study, the real-time analysis of fluorescent interneurons in hippocampal organotypic cultures showed that the appearance rate of new spines was significantly increased 24 h after the addition of Endo-N to the culture medium. However, a strong increase in the relative spine density in the slices treated with Endo-N was found only when the cultures were incubated with Endo-N for 48 h (Guirado et al., 2014a). When this parameter was analyzed after 24 h of Endo-N treatment a tendency for a decrease was observed. It is possible that these differences are due to the distinct phenotype of EGFP–labeled cells between the hippocampus and the mPFC, differences in the culture media and/or the methodology used for the estimation of the spine density in these two studies. Moreover, the estimation of this density in hippocampal interneurons in real time was performed in a central portion of the dendrites. It also has to be noted that the spine density in the interneurons of organotypic cultures is almost twice as high than in those of the adult, both in the hippocampus and in the mPFC (Guirado et al., 2014a,b and present results).

Previous work on EGFP-expressing spiny neurons in the mPFC of adult GIN mice has revealed that these cells are Martinotti cells. Although in this mice strain a subpopulation of EGFP expressing hippocampal interneurons co-expresses PSA-NCAM, in the mPFC these fluorescent interneurons do not show PSA-NCAM expression in their somata, neurites or in the puncta located in their projection fields in layers I and II *in vivo* or in organotypic cultures (Oliva et al., 2000; Nacher et al., 2013; Guirado et al., 2014a). It is possible that this lack of PSA-NCAM expression might be due to a putative competition of synthetic pathways between EGFP and PSA-NCAM, since, at least in the adult rat mPFC, a subpopulation of Martinotti cells expresses PSA-NCAM (Gómez-Climent et al., 2011). The effects of PSA depletion might have thus to operate through an indirect pathway to induce the changes in dendritic spine density observed in our study. It is, however, important to note that the scenarios in which these EndoN-induced changes in interneuronal spines are occurring may be different between the organotypic cultures and the adult mPFC: although PSA-NCAM levels are already strongly downregulated after 11–15 DIV, they are higher than those in the adult mPFC. Moreover, in perinatal animals, PSA-NCAM is still expressed by some neuronal populations, which do not express it in adults (Bonfanti, 2006). We have shown here that, in the organotypic cultures used in this study, PSA-NCAM is present in the somata of CB expressing interneurons, many of which also express PSA-NCAM in the mPFC of adult animals. However, we have also found PSA-NCAM immunoreactivity, although very reduced, in the somata of CR and PV expressing interneurons. These two subpopulations rarely express PSA-NCAM in their somata in the mPFC of adult animals, although many PV expressing puncta on pyramidal neurons express this molecule (for review see Bonfanti, 2006; Nacher et al., 2013). In any case, it would be interesting to exclude non-enzymatic effects of EndoN on the interneurons lacking PSA expression by using inactivated forms of the enzyme.

If PSA-NCAM depletion is not directly involved in the spine remodeling observed in Martinotti EGFP labeled interneurons, what may be the mechanism producing this changes in spine density? Our present results show an increase in the expression of VGLUT1 and SYN in the mPFC after PSA depletion, indicating that this treatment may induce an increase in the number of excitatory synapses. Probably these synapses were not still fully functional in the cultures because of the presence of PSA-NCAM, and the ablation of this molecule may have promoted their final maturation. Although certainly the proportion of excitatory synapses located on interneuronal spines is very reduced when compared to the total number in the mPFC, some of the new excitatory synapses induced by Endo-N may contact these spines. Two recent studies have described that, in the visual cortex and the CA1 region of the hippocampus, interneuronal spines receive mainly a glutamatergic input (Keck et al., 2011; Guirado et al., 2014a). Consequently, it is possible that many of these new glutamatergic synapses have been formed on new spines developed in the proximal region of EGFP-labeled interneuronal dendrites. This increase in excitatory input on this region would increase their inhibition on pyramidal neurons and may act as a compensatory mechanism to reduce the alteration in the E/I ratio. However, the scenario has to be more complex, since the loss of spines in the distal dendritic segments would lead to the opposite effects. Obviously these changes in dendritic spine density may play an important role in the activity-dependent modulation of neuronal connectivity within local cortical circuits, as already has been suggested (Chen and Nedivi, 2010), but their significance will be far from clear until future studies analyze the connectivity of the spines in the different segments.

### Changes on the Perisomatic Puncta Around Pyramidal Neurons

PSA-NCAM expressing puncta have been found surrounding the somata of mPFC pyramidal neurons in adult rats and mice (Castillo-Gómez et al., 2011; Guirado et al., 2014a). Around one third of these puncta co-expressed markers of inhibitory elements or synapses, such as GAD65/67 or VGAT and rarely co-expressed VGLUT1, a marker of excitatory synapses, indicating that most of these puncta correspond to inhibitory synapses. In fact, the synaptic vesicle protein SYN was also found in approximately one third of PSA-NCAM expressing puncta (Castillo-Gómez et al., 2011). Most PSA-NCAM expressing perisomatic puncta co-expressed PV, but rarely CR or CB, suggesting that they belong to basket/fast spiking neurons. Interestingly, these basket cells do not express PSA-NCAM in their somata, but only in their terminal boutons surrounding pyramidal neurons (Castillo-Gómez et al., 2011). The results found after Endo-N treatment in the present study using mPFC organotypic cultures are very similar to those observed after *in vivo* depletion of PSA using intracerebral injections of the enzyme (Castillo-Gómez et al., 2011). Endo-N treatment *in vivo* increased the density of inhibitory perisomatic puncta expressing GAD65/67 or PV, which was accompanied by a parallel increase in the density of those expressing SYN and of those co-expressing PV and SYN. In the present study, we have also found increases in the perisomatic density of puncta expressing the inhibitory markers GAD-EGFP, VGAT and PV, as well as in SYN, suggesting that, as it appears to occur in *vivo*, PSA depletion promotes the appearance or maturation of functional inhibitory synapses around the somata of mPFC pyramidal neurons. This suggests an important role for PSA-NCAM in the control of the plasticity and stability of the perisomatic inhibitory innervation of pyramidal neurons in the mPFC. The presence of PSA on NCAM produces a steric impediment for homotypic and heterotypic binding (Johnson et al., 2005) and consequently, its presence on some perisomatic synapses may produce an “insulating effect”, inactivating these contacts (Castillo-Gómez et al., 2011; Nacher et al., 2013). Therefore, the removal of PSA induced by Endo-N may promote the activation of previously silent inhibitory synapses, increasing the expression of molecules related to active inhibitory neurotransmission, such as SYN, GAD and VGAT, making more puncta detectable. In agreement with these observations *in vivo* and *in vitro*, a previous study on the developing visual cortex found that PSA depletion induced the precocious maturation of perisomatic innervation by basket interneurons, resulting in enhanced inhibitory neurotransmission (Di Cristo et al., 2007). It is interesting to note that the density of perisomatic puncta expressing the excitatory synaptic marker VGLUT1 on mPFC pyramidal neurons is also increased after PSA depletion in organotypic cultures. The density of these excitatory puncta is extremely low when compared to that of inhibitory ones in the adult mPFC (Castillo-Gómez et al., 2011). However, in the organotypic cultures their density appears to be similar. We still do not understand the nature, the fate or the functional significance of these perisomatic puncta expressing excitatory markers. It is commonly believed that pyramidal cell somata receive only symmetric (inhibitory) synapses from axon terminals of GABAergic interneurons (Merchán-Pérez et al., 2009). Future studies should be directed to know whether the VGLUT1 expressing puncta on the perisomatic region of pyramidal cells are functional excitatory synapses. The unchanged E/I ratio of the perisomatic region is most likely due to the parallel increase in the density of both inhibitory and excitatory puncta in this region.

The mechanisms by which PSA-NCAM may regulate the perisomatic innervation of pyramidal neurons, apart from exerting a steric impediment for the proper function of synapses expressing this molecule, are still unclear. However, other factors, such as the facilitation of BDNF signaling on interneurons by PSA depletion, may also be involved (Gorba and Wahle, 1999; Berghuis et al., 2004; Fiorentino et al., 2009).

### Putative Implication of the Present Results in Schizophrenia and Major Depression

The finding of this prominent role of PSA-NCAM in the regulation of the perisomatic innervation of pyramidal neurons by basket cells and in the alteration of the E/I ratio in the neuropil of the mPFC may have important implications for our understanding of the etiopathology of schizophrenia and major depression. Both NCAM and one of the enzymes that transfer PSA to this adhesion molecule (ST8SiaII) are susceptibility genes for schizophrenia and changes in the expression of PSA-NCAM have been observed in the PFC cortex of schizophrenic and major depression patients (for review see Nacher et al., 2013; Wędzony et al., 2013). However, in the PFC of schizophrenia patients the density of PV-immunoreactive puncta and the expression of mRNA and protein for GAD67 in PV expressing interneurons are reduced (for review see Lewis et al., 2011; Marín, 2012). Moreover, the levels of PV protein are lower in the pyramidal perisomatic boutons formed by basket cells (Lewis et al., 2011). Together, these results indicate that the density and/or strength of the perisomatic inputs of PV expressing basket cells to pyramidal neurons is decreased in the PFC of schizophrenic patients. This is in conflict with our present results, which describe an increase in the density of perisomatic inhibitory puncta surrounding pyramidal neurons after PSA depletion. This discrepancy may be explained by the fact that our results are obtained after an acute and complete depletion of PSA, while the decrease in PSA-NCAM expression in schizophrenic patients may be a much longer process, which probably starts during early development. Such a prolonged time course may have induced compensatory changes in the PFC circuitry. In addition, it has to be noted that in the studies of PSA-NCAM expression in the PFC most psychiatric patients were subjected to chronic treatments with typical or atypical antipsychotics or antidepressants. These treatments may have an important impact, because antidepressants increase the expression of PSA-NCAM and the antipsychotic haloperidol decreases this expression in the rodent mPFC (Nacher et al., 2013; Wędzony et al., 2013; Guirado et al., 2014b).

Disruption of the E/I balance in the PFC may play a key role in schizophrenia (Yizhar et al., 2011). There is ample evidence supporting the hypothesis that prefrontocortical disinhibition occurs in this disorder, due to the disrupted functioning of inhibitory interneurons, which results in an elevated E/I ratio (for review see Marín, 2012; Inan et al., 2013). The depletion of PSA in our organotypic mPFC cultures reproduces this scenario and may constitute a valuable tool for investigating the causes and consequences of this alteration and a good experimental approach to increase our knowledge on the etiopathology of schizophrenia.

## Author Contributions

EC-G and JN designed the study; EC-G, MP-R and SV performed the experiments; EC-G analyzed the data; EC-G and MP-R prepared the figures; EC-G and JN wrote the manuscript.

## Funding

This work was supported by Spanish Ministry of Economy and Competitiveness (BFU2012-32512), Generalitat Valenciana (Prometeo Excellence Program PROMETEO2013/069) and the Fundación Alicia Koplowitz to JN. MP-R had a FPU predoctoral fellowship from the Spanish Ministry of Economy and Competitiviness (FPU12/03200).

## Conflict of Interest Statement

The authors declare that the research was conducted in the absence of any commercial or financial relationships that could be construed as a potential conflict of interest.
